# Reaching out to patients with intellectual disabilities

**Published:** 2013

Worldwide, 1.04percnt; of the population is estimated to have an intellectual disability; however, this proportion has been found to be higher in low- and middle-income countries.^1^

According to the WHO, ‘intellectual disability’ is defined as ‘a significantly reduced ability to understand new or complex information and to learn and apply new skills’. This results in a reduced ability to cope independently, which begins before adulthood and has a lasting effect on development.

In many poor or remote communities, people with intellectual disabilities are stigmatised and their families may become isolated. Outreach and community services have an important role to play in ensuring that people with intellectual disabilities get access to the eye care they need.

People with intellectual impairments may have a higher than average prevalence of visual impairment. Sight problems may also result from brain damage, cerebral visual impairment, or may be associated with other causes of intellectual impairment.^2^ Individuals with intellectual impairments may have to be reminded about how to take care of their spectacles and about the importance of eye tests, and they may need support in order to attend for tests.

People with intellectual impairments may struggle to get the same quality of care when they do come forward for medical treatment. In the UK, the health outcomes of people with intellectual disabilities are poor, and some have died unnecessarily in hospital because of failures to understand and address their particular needs.^3^

## What must eye care workers know?

- People with learning difficulties may find it difficult to understand complex instructions or questions. They may struggle to understand the consequences their decisions may have on their health.
- They may find it difficult to make decisions and will take longer to do so.
- Some people with learning disabilities may struggle with reading and writing and may have additional challenges such as physical and/or sensory impairments.
- Health problems might be accompanied by unusual signs and symptoms – for example, someone with severe learning disabilities might demonstrate discomfort by self-harming.
- People with learning disabilities often rely on family members and carers to provide emotional support and to help them feel safe.
- There might be barriers to attending health services, such as poor physical access, transport costs, difficulties in finding their way around, or having no-one to accompany the person.

## Improving our service

Simple, cost-effective changes can make a big difference in our ability to provide quality care to people with intellectual disabilities. Here are some examples.

**General**

- Allow more time for people with intellectual impairments. For example, book double appointments, or give them either the first or the last appointment of the day.
- Make sure that visiting hour restrictions do not apply to carers.
- Provide a bed or chair for carers or family members so they can stay overnight if needed. If the patient receives food and drink, offer the same to their carer or family member.
- Treat people with intellectual disabilities – and their families and carers – with dignity, kindness and respect.

**Figure:**
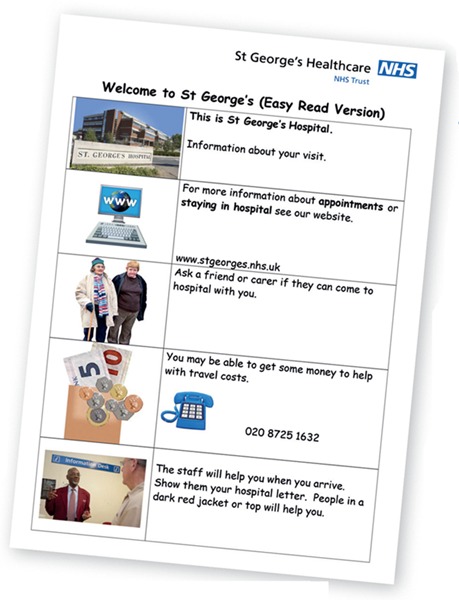
‘Easy read’ information for people with an intellectual impairment. UNITED KINGDOM

**Communication**

- Always speak to people with intellectual disabilities first, not the person supporting them. Take time to listen to their response.
- If they have difficulty answering questions then ask the person supporting them – but remember they may have different views from each other.
- Introduce yourself and explain your role: give your name and speak clearly, in short sentences, and not too fast.
- Communicate with the person with an intellectual disability by using visual aids (such as illustrations, photographs, pointing to objects), and using clear and simple language. Do not use abbreviations or jargon!
- Always explain at each stage of the examination or procedure what will happen next. People with intellectual disabilities do not like surprises.
- It may be helpful to create ‘easy read’^4^ guidance to the hospital and common procedures (see illustration); these are written very simply, in a larger font, and accompanied by clear pictures (one picture or symbol per idea). This will save time in future.

**Environment**

- Make sure that lighting is not too bright or intrusive.
- Sudden noise can be very stressful. Keep noise to a minimum, or take the patient to a quieter area. Noise (announcements, television, or radio) can be very distracting.
- Keep the environment clean and tidy. Too much clutter can distract people and make it difficult for them to visually focus on you.
- Make sure that people know where the bathroom facilities and waiting room are, and that these areas are well signposted.

Compiled by Elmien Wolvaardt Ellison
